# Social Connectedness and Loneliness Among U.S. Older Adults During the COVID-19 Pandemic

**DOI:** 10.1093/geroni/igaf122.3122

**Published:** 2025-12-31

**Authors:** Qiyu Deng

**Affiliations:** Syracuse University, Syracuse, New York, United States

## Abstract

Using data (N = 4,261) from the Health and Retirement Study (HRS) 2020, this study examines how pandemic-related social connectedness associates with loneliness among U.S. older adults. Pandemic-related social connectedness was measured by a composite indicator integrating social network size, frequency of interactions with children and other family members, and instrumental and emotional support provided and received outside the household. Loneliness was operationalized as a binary outcome using UCLA 3-item scale. Logistic regression incorporating sampling weights was estimated, adjusting for age, gender, race, marital status, living arrangement, functional limitations, self-rated health, depression, education, household size, household income, employment status, and urbanicity. Results showed that greater social connectedness significantly lowered the odds of loneliness (OR = 0.79; p = 0.01). Older adults aged 65 to 79 and those aged 80+ reported lower odds of loneliness compared to adults aged 50 to 64. Moderate and severe functional limitations were significantly associated with higher loneliness odds (OR = 1.49 and OR = 1.68 respectively, p < 0.01). Depression was strongly predictive of loneliness (OR = 1.33–4.58, p < 0.001). Adults living with others, but unmarried or unpartnered, had substantially higher odds of loneliness compared to married or partnered adults (OR = 2.14, p = 0.002). Household size, household income, race, self-rated health, education, employment status, and geographic location were not significantly associated with loneliness. These findings highlight that older adults experiencing functional limitations and depressive symptoms may be particularly vulnerable to loneliness, emphasizing the need for targeted support and interventions to enhance social connectedness during challenging times such as the COVID-19 pandemic.

